# Percutaneous dilational tracheotomy in liver transplant recipients

**DOI:** 10.1186/2197-425X-3-S1-A905

**Published:** 2015-10-01

**Authors:** A Kundakci Ozdemirkan, Z Ersoy, P Zeyneloglu, E Gedik, A Pirat, M Haberal

**Affiliations:** Baskent University, Ankara, Turkey

## Introduction

Liver transplant recipients (LTR) may require percutaneous dilational tracheotomy (PDT) during the immediate postoperative period or later because of need for prolonged mechanical ventilation or airway issues. However, despite the increased risk of bleeding and infections, there is little data regarding the safety and effectiveness of PDT in LTRs.

## Objectives

The aim of this study was to evaluate the safety and effectiveness, in terms of changes in oxygenation and lung compliance, of PDT in LTRs.

## Methods

We reviewed the data of liver transplant recipients who underwent percutaneous dilational tracheotomy in Baskent University Hospital between January 2010 and March 2015. Collected data included demographics (age, sex, body weight, length); etiology of chronic liver failure; comorbidities; Child, Model for End Stage Liver Disease (MELD), acute physiology and chronic health evaluation II (APACHE II), and sequential organ failure assessment (SOFA) scores; length of hospitalization and mechanical ventilation; etiology of acute respiratory failure; pre-PDTPlatelet count and international normalized ratio (INR) values; partial pressure of arterial oxygen to fractional inspired oxygen ratio (P/F), and pulmonary compliance. Pre- and post-PDT values were compared using Wilcoxon test.

## Results

Out of 136 LTRs 16 required PDT during the study period. All PDTs were performed by experienced intensivists and under bronchoscopic guidance using Percutwist or Ciaglia techniques. The mean age was 35.4 ± 16.5 years and mean body mass index was 25.5 ± 6.0 kg/m^2^. The mean Child and MELD scores were 10.5 ± 1.9 and 23.5 ± 7.7, respectively. The mean APACHE II on admission was 25.4 ± 11.9 Pre-PDT platelet count and INR were 47000 ± 41000 per µl and 2.0 ± 0.8, respectively. The indication for PDT was prolonged mechanical ventilation for all patients. The etiology of acute respiratory failure was most commonly extrapulmonary (%87.5). The mean interval from transplantation to PDT was 355 ± 870 days. The only major complication noted was left-sided pneumothorax in one patient. Six patients had minor, sel-limiting bleeding from the tracheotomy site on the first day of post-PDT. There were no significant differences between pre-PDT and post-PDT P/F ratios (261 ± 203 vs 264 ± 132; p:0.534). However pre- and post-PDT pulmonary compliances were significantly different (0.02 ± 0.02 L/cmH_2_O vs 0.03 ± 0.02 L/cmH_2_O, p:0.002). The number of patients who required sedation significantly decreased after PDT (7 versus 1, p = 0.03).

## Conclusions

When performed by experienced intensivists using bronchoscopic guidance PDT is safe in LTR. PDT may also improve lung mechanics and decrease the need of sedation in these patients.

